# Youth participation in public health: from presence to power

**DOI:** 10.1093/eurpub/ckaf250

**Published:** 2026-02-17

**Authors:** Tit Albreht, Charlotte Marchandise, Hans Henri P Kluge, Olha Izhyk, Cristiana Salvi, Katrine Bach Habersaat, Manuel Franco, Jennie Elzinga

**Affiliations:** President of EUPHA; Executive Director of EUPHA; WHO Regional Director for Europe; WHO Regional Office for Europe; WHO Regional Office for Europe; WHO Regional Office for Europe; Chair of the 19th EPH Conference; Conference Manager EPH Conference

Across Europe, the need to strengthen social participation is increasingly recognised as a core component of public health governance. The recent WHO Resolution on Social Participation for Universal Health Coverage (WHA77, 2024) formalises this shift, urging health systems to establish sustained, structured, and accountable mechanisms enabling communities to influence decisions. Yet one group remains inadequately engaged despite being profoundly affected by today’s health challenges: young people.

## Participation as a governance function, not a gesture

Classic theories such as Arnstein’s ‘Ladder of Citizen Participation’ highlight the distinction between symbolic inclusion and shared decision-making. Much youth engagement still sits in the lower rungs of this ladder. Feedback is collected, but impact on the policy cycle remains minimal. Meaningful participation requires investment in public-sector capacity, clarity of purpose, and stable institutional architecture. These requirements apply even more strongly when engaging adolescents and early-career professionals, whose presence is often welcomed but rarely supported through formal governance pathways.

## Why youth participation matters: evidence over symbolism

The case for youth involvement is well-established. Public health research shows that integrating youth perspectives increases policy relevance, strengthens trust, and improves responses to rapidly evolving determinants such as digital environments, climate anxiety, sexuality, and mental health. The United Nations definition of meaningful youth engagement emphasizes autonomy, influence, and co-responsibility, elements often missing from existing frameworks.

Youth participation is also an equity question. Young people disproportionately experience social and health inequalities, from precarious employment to mental health burdens. Their lived experience constitutes expertise in its own right, complementing quantitative data and professional analysis.

When young people are excluded from the spaces where social and societal issues are debated, this has consequences for their wellbeing. It affects their sense of agency and belonging, fuels frustration and disengagement, and weakens trust in institutions, factors closely linked to mental and social health outcomes. Ensuring meaningful participation is therefore not only a governance principle, but also a preventive measure: it strengthens resilience, reduces polarisation, and supports the conditions under which young people can thrive. In this sense, participation itself becomes a determinant of health.

## Evidence from research: participation improves science

Participatory and co-production approaches have reshaped public health research. Studies have demonstrated that when communities- especially young people- co-design research tools and priorities, outcomes are more relevant and implementation more effective. EUPHA is also partner of the EARLY project (EARLY (evaluating, identifying and reducing determinants of mental health conditions in youth)—The European Public Health Association), that aims at providing robust and representative data on youth mental health across the European region, and to design, implement, and evaluate evidence-based interventions. But we need to do better, not to do for youth, without them. The recent French MENTALO (MENTALO. https://etude-mentalo.fr/) study illustrates this approach: co-developed with young people, it reached unprecedented participation and produced insights grounded in the realities of youth mental health. This model aligns with the growing shift toward participatory epidemiology, youth advisory boards, and community-led indicators.

## Barriers persist, and they are systemic

Across literature on participatory governance, recurring barriers include lack of methodological guidance, weak institutional legitimacy, and structural power imbalances. Youth engagement faces additional challenges stemming from adult-centric institutional cultures and limited adaptation to youth-preferred communication channels. Importantly, these obstacles rarely originate from young people themselves but from governance architectures not designed for their participation.

## Strengthening structures: the role of EUPHAnxt

Within the European context, one emerging structure designed to address these gaps is EUPHAnxt, the network for students and early-career professionals in public health. Beyond offering training, mentoring, and conference support, EUPHAnxt provides a platform that aligns with the principles of meaningful participation: continuity, capacity-building, and shared ownership. Its involvement in conference planning, plenary design, scientific sessions, and mentoring activities demonstrates a shift from ad hoc youth inclusion toward structured, institutionalised engagement.

However, as participation research reminds us, visibility and transparency are essential components of legitimacy. When youth contributions are embedded early in processes—co-designing programme content, shaping narratives, contributing scientific expertise- their influence may be substantial yet insufficiently communicated to the wider community. Strengthening communication about youth and early-career involvement, and clarifying the governance roles they occupy, may therefore enhance external recognition of their contribution and address perceptions of insufficient representation. This is consistent with the broader evidence that participation must be both meaningful and visibly acknowledged to generate trust and accountability.

## Towards a new generation of participation in Europe

EUPHAnxt’s expanding role, and emerging youth-led networks across the WHO European Region collectively demonstrate a shift toward co-production in public health. The challenge ahead is not to involve young people more often, but to embed their participation into the governance, research, and implementation cycles in ways that are deliberative, transparent, and institutionally supported.

As Europe faces intertwined crises, from mental health to climate to digital misinformation, youth participation cannot remain an accessory to public health governance. It is a necessary condition for developing more equitable, resilient, and legitimate health systems.

## False health information: what’s really at stake

KlugeHans Henri P
WHO Regional Director for Europe
IzhykOlha
WHO Regional Office for Europe
SalviCristiana
WHO Regional Office for Europe
HabersaatKatrine Bach
WHO Regional Office for Europe

Fatigue, anxiety, politicisation, and an endless flood of online falsehoods have worn away at people’s confidence and trust in science: our compass for navigating health crises. That’s why it is vital to remember that the loudest voices undermining science, are *not* the majority. Surveys covering over 70 000 people in 68 countries, including 31 across the WHO European Region, show that most people still trust scientists–and want them to play a stronger role in shaping policy (Nature in human behaviour).

The question then becomes, how do we deliver that? First of all we need to acknowledge that mis- and disinformation is not a passing storm. It is a reality that we need to meet with skill, political will and investment.

### Our new reality

Studies show that up to half of vaccine-related posts on social media contain mis- or disinformation, and nearly a third of pandemic-related content is misleading or false (Borges do Nascimento IJ, Pizarro AB. Infodemics and health misinformation: a systematic review of reviews. Bull World Health Organ. 2022 Jun 30;100(9):544–561). False health claims are not new, but the magnitude and volume are for a number of reasons, the age of social media and artificial intelligence being one. Today, false health claims are the background noise to our lives, and that constant presence makes it easy to feed harmful narratives, and hard to stop them.

We have seen how false information, amplified by influential figures can travel fast and sow confusion, and to give just one example, prompt parents to delay or refuse vaccinations. Small but vocal minorities then magnify doubt.

The fact is that in today’s world, mis- and disinformation is a chronic risk. And because false information doesn’t appear in a vacuum but grows out of unanswered questions, lack of timely, clear information and low trust–our ability to prepare and respond must match that reality.

Infodemic management must be a constant, fully integrated into preparedness and response. That means building resilience, targeting those known to have low health literacy, and equipping health systems to address misperceptions respectfully and effectively. Because protecting the information environment is a public health priority, as fundamental as vaccination campaigns or disease surveillance.

### The fallout

False health information has a direct impact on public health. A 2020 study estimated that false online claims led to thousands of hospitalizations and hundreds of deaths in the first months of the COVID-19 pandemic (World Health Organisation. *Fighting misinformation in the time of COVID-19, one click at a time.*  https://www.who.int/news-room/feature-stories/detail/fighting-misinformation-in-the-time-of-covid-19-one-click-at-a-time#:∼:text=Acting%20on%20the%20wrong%20information, 19).

But the damage goes far beyond individual harm. What’s at stake is the long-time erosion of trust. That is why mis- and disinformation ranks third in the UN’s 2024 Global Risk Report (UN Global Risk Report. https://unglobalriskreport.org).

Trust in science is fragile: hard to build, easy to lose, vital to protect. Falsehoods fracture communities, fuel fear, and corrode trust in institutions meant to keep us safe. Falsehoods weaken social fabric, divide communities, encourage polarization, and erode solidarity. And once trust in science collapses, even the best of policies loses their impact.

While trust is shaped by culture and context, certain factors consistently contribute to it. Collaboration, open dialogue and transparency to name a few, on which Denmark, as an example, knows a thing or two. During the COVID-19 pandemic, Denmark applied a ‘radical transparency’ approach that saw the public informed consistently and openly. This approach is credited with their successful disease suppression and high public compliance to public health and social measures.

### Building resilience

Most governments, media platforms, and civil society have learned the lessons.

Across the WHO European Region, their efforts are growing to improve fact-checking, make reliable information accessible, provide clear and timely corrections in times of uncertainty, and strengthen critical thinking. In other words, boosting public immunity to harmful, misleading information by strengthening digital health literacy. Scotland, to give just one example, recently released their first *Health Information Integrity Strategy–*a national approach to addressing false health information that is fact-based, ethical, coherent.

Different approaches help people spot false information. And as in the case of vaccines, information inoculation of this sort—needs to be timely. Sustained investment in resilience of both the public and health systems alike—is key.

### Our defence

Under the Second European Programme of Work 2026–2030, WHO/Europe has made addressing mis- and disinformation a priority. With evidence, transparency and empathy, we seek to address false, misleading health claims.

In the past year, WHO/Europe has developed several tools to support Member states in managing infodemics, step by step. We have trained more than 1,500 officials to detect and respond to false information–from monitoring and fact-checking to engaging community actors in delivering accurate health advice. We launched a school-based education programme against vaccine-misinformation, soon available in 14 languages. We continuously review data on vaccine efficacy and safety, and update recommendations.

Looking ahead, we plan to strengthen our work on helping people identify and resist false information early. We are also exploring how artificial intelligence can support, responsibly yet effectively.

The future of public health depends not just on scientific progress, but on people’s belief in it. Because if people don’t trust science or the health advice they receive–even the strongest health system will falter. So our best defence isn’t just faster, better vaccines, treatments or tests. It’s trust in them–built through openness, honesty, and shared purpose. *This is WHO/Europe’s last regular editorial for EJPH, that have covered topics ranging from healthy ageing and digitization of health systems—to antimicrobial resistance and the health impact of climate change. We look forward to continuing the collaboration and dialogue on an ad-hoc basis in future issues.*

## Urban and global synergies: shaping the future of public health with climate resilience, equity and innovation

FrancoManuel
Chair of the 19th EPH Conference
ElzingaJennie
Conference Manager EPH Conference

### Welcome to bilbao, a city shaped by continuous transformation

Following the highly successful conference in Helsinki, we are delighted to invite you to the 19th European Public Health (EPH) Conference, taking place from 10 to 13 November 2026 in Bilbao, Spain. The Euskalduna Conference Centre will host this year’s edition, set in a city that has undergone one of Europe’s most remarkable urban transformations. Once an industrial hub along the Nervión river, Bilbao has reinvented itself as a centre for culture, design, active mobility and sustainable urban renewal. This vibrant city offers an ideal backdrop to reconnect with colleagues, exchange ideas, and explore the future directions of public health in Europe and worldwide.

### Main theme: urban and global synergies

The overarching theme of the 19th EPH Conference–*Urban and global synergies: shaping the future of public health with climate resilience, equity and innovation–*reflects the profound and interlinked challenges facing population health today. Urbanisation is accelerating worldwide, with two-thirds of the global population projected to live in cities by 2050. In Europe, this figure already stands at 80%. Cities concentrate opportunities, resources, and innovation, but they also reveal deep social inequalities and increasing environmental and health stressors.

The conference theme underscores the need to rethink how public health research and policy can support thriving, equitable and climate-resilient urban environments. Addressing health inequities, integrating planetary health perspectives, and fostering evidence-informed action are essential to shaping policies that meet the needs of both current and future generations.

#### Subthemes

The conference in Bilbao brings together researchers, practitioners, policymakers and educators from different places around the world working at the intersection of population health, climate change, equity, and urban development. This year’s programme will explore the following interconnected subthemes:

- How do we survive climate change?
   Climate-related health risks–heatwaves, floods, displacement and extreme weather events–are growing rapidly. Understanding and adapting to these threats and impacts is central to protecting population health.
- The city as a catalyst for change
   Cities can both challenge and improve health. Approaching urban health through the lens of raising inequalities, social determinants and environmental exposures opens new avenues for public health research and innovation.
- Providing equity in urban and rural settings
   Insights from urban health research can also inform approaches to rural health challenges and vice versa. Both contexts are interdependent requiring connected strategies.
- Health by design
   Urban planning, urban mobility systems, housing, and public spaces design and use shape health. Designing environments that promote well-being is a vital interdisciplinary effort.
- Public health diplomacy and conflicts: the fight for hope
   As global norms and institutional frameworks come under pressure, public health diplomacy plays a crucial role in fostering peace and international collaboration to health protection.

Together, these subthemes highlight the importance of interdisciplinary collaboration and innovative data and research approaches, enabling meaningful translation of evidence into policies that improve health and reduce inequities in Europe and worldwide.

### Bilbao as host city

Bilbao offers an inspiring setting for the 19th EPH Conference. Its reinvention has prioritised accessible public spaces, green corridors, cultural vitality, and sustainable mobility. From the urban connection of highly diverse neighbourhoods, to Guggenheim Museum’s riverside urban renewal, to the intimate charm of the Casco Viejo, the city reflects how urban design can enhance quality of life while embracing diversity and human and planetary sustainable development. Bilbao’s compact scale and scenic surroundings provide a welcoming environment for conference delegates to explore and connect.

Under the leadership of Conference Chair Manuel Franco, and supported by a strong Spanish and Basque public health community, Bilbao 2026 promises a rich scientific programme and a stimulating atmosphere for public health professionals from across Europe and beyond.

We look forward to welcoming you to Bilbao–**Ongi etorri Bilbora!**

